# Anatomy: Descriptive and Surgical

**Published:** 1883-09

**Authors:** 


**Anatomy: Descriptive and Surgical.** By HENRY GRAY, F. R. S., Fellow of the Royal College of Surgeons, etc. With an introduction on General Anatomy and Development, by T. HOLMES, M. D., Surgeon to St. George's Hospital. The drawings are by H. V. CARTER, M. D. A new American, from the tenth English edition, to which is added: Landmarks, Medical and Surgical, by LUTHER HOLDEN, F. R. C. S., with additions by WM. W. KEENE, M. D. Philadelphia: H. C. Lea's Son & Co. 1883.

This new edition of Gray's Anatomy needs only to be announced. There is hardly a physician or even a student in the land who is not familiar with "old Gray." The physician, however, who studied medicine in the "sixties," and who has been so content with Gray's Anatomy of that date as not to have looked into succeeding editions, would open his eyes at the progress of the science as evidenced in this present edition. It is now, indeed, a grand work, containing over 1,000 pages. Many chapters have been entirely rewritten and much new matter and many illustrations have been introduced. Lea's

Son & Co.'s work is always unexceptional; and this work, both as to paper, letter-press and binding, is an instance of perfection of book-making.

